# Sepsis Associated With Aggressive Cutaneous Angiosarcoma

**DOI:** 10.7759/cureus.55619

**Published:** 2024-03-06

**Authors:** Victoria Palmer, Trie Arni Djunadi, Bhuvana Tantry, Angela Grigos

**Affiliations:** 1 Internal Medicine, Richmond University Medical Center, Staten Island, USA

**Keywords:** cutaneous angiosarcoma, dermatopathology, skin cancer, cutaneous malignancy, sepsis

## Abstract

Cutaneous angiosarcoma is an aggressive tumor most commonly presenting on the head and neck. In this case report, we describe the presentation of sepsis secondary to an aggressive and rapidly expanding wound, located in a sun-protected area on the body, in a patient with multiple concurrent comorbidities. Treatment was tailored toward targeting the causative organisms, as well as identifying the histologic morphology of the pathologic legion. Histopathology and immunohistochemistry were used to confirm the diagnosis of cutaneous angiosarcoma, and the patient-centered decision surrounding palliative chemotherapy is outlined.

## Introduction

Cutaneous angiosarcoma (CA) stands out as the predominant and aggressive form of tumor, constituting approximately 1% of all soft tissue tumors, with an incidence rate ranging from 0.01 to 0.05 per 100,000 individuals annually [1]. The incidence rate of CA experiences a noteworthy escalation, ranging from 0.03 to 2.25 per 100,000 in patients with antecedent primary cancer. Predominantly afflicting the head and neck, followed by the breast, visceral regions, and other cutaneous sites, CA manifests with a distinctive gender predisposition, affecting men more frequently than women in a ratio of 2:1. Additionally, individuals with lighter skin types exhibit a heightened susceptibility and have greater documentation in the literature [2]. This case report aims to provide a brief overview of an unusual presentation of cutaneous angiosarcoma. Additionally, the aggressive nature of the disease entity as well as the associated complication of sepsis serves as a learning tool for physicians and trainees in dermatology, primary care, and hospital medicine.

## Case presentation

**Figure 1:**
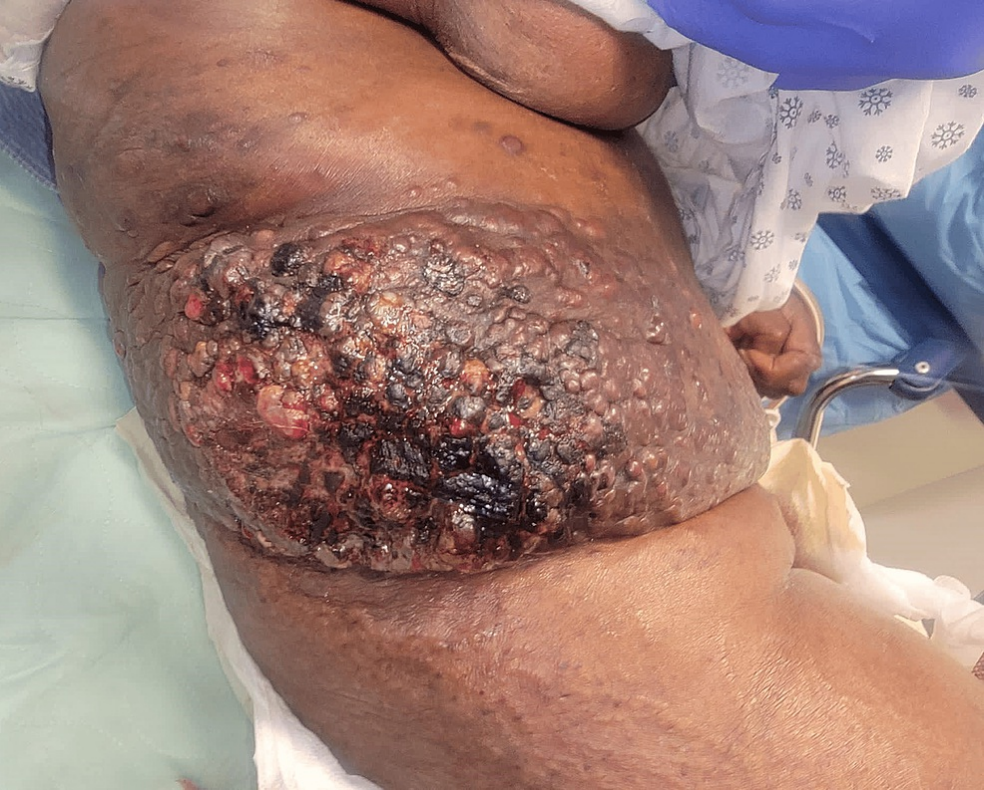
Demonstration of the violaceous, indurated, and ulcerating plaques and nodules involving the right flank and right mid-lower quadrant of the abdomen. Edema of the surrounding tissue is also visible.

**Figure 2:**
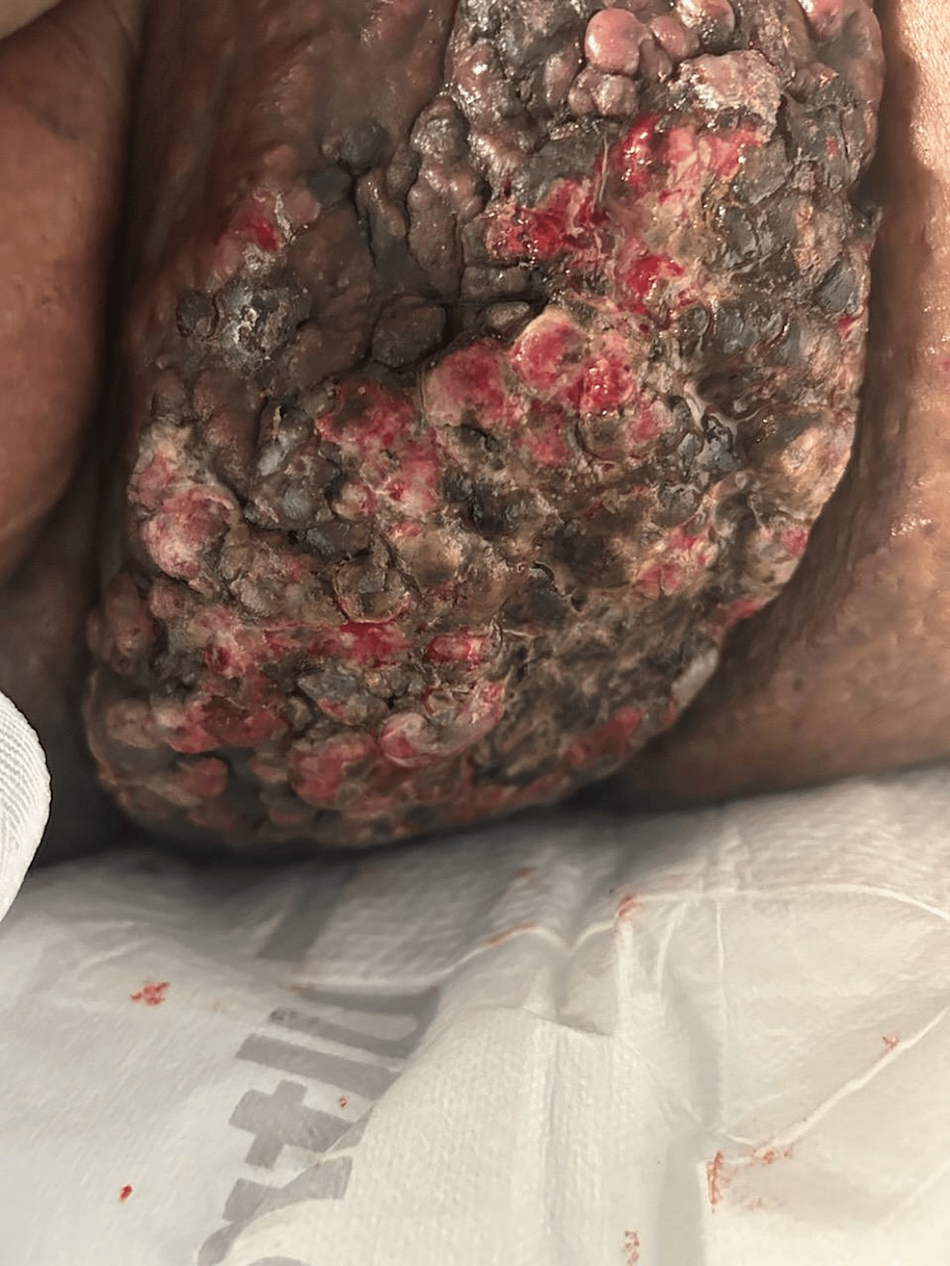
A closeup of the lesion Cobblestone appearance and purulent drainage are noted.

We present a 77-year-old female with a past medical history of hypertension, atrial fibrillation, heart failure with preserved ejection fraction, cerebrovascular attack, and myocardial infarction with anoxic brain injury who was brought into the hospital by her nursing home facility due to an asymptomatic enlarging malodorous mass to the right flank over approximately three months. On presentation, the patient was tachycardic, febrile, and had an elevated white blood cell count of 14,000 x 109/L, thus meeting the systemic inflammatory response (SIRS) criteria for sepsis. On examination, the patient had extensive indurated, violaceous, and ulcerating plaques and nodules involving the right mid-lower quadrant of the abdomen and extending to the right flank (Figures 1, 2).

The patient had no prior history of surgery, radiation, or lymphedema in this area. Previous trials of mupirocin ointment, as well as oral doxycycline and ciprofloxacin, were unsuccessful. The patient received a course of aztreonam, vancomycin, and acyclovir while in the hospital as the initial differential diagnoses included infected varicella zoster or infected pyoderma gangrenosum (for which oral prednisone 40 mg daily was later started). Further differential diagnoses included cutaneous T or B cell lymphoma; however, a leukemia/lymphoma panel was performed and results were negative.

**Figure 3:**
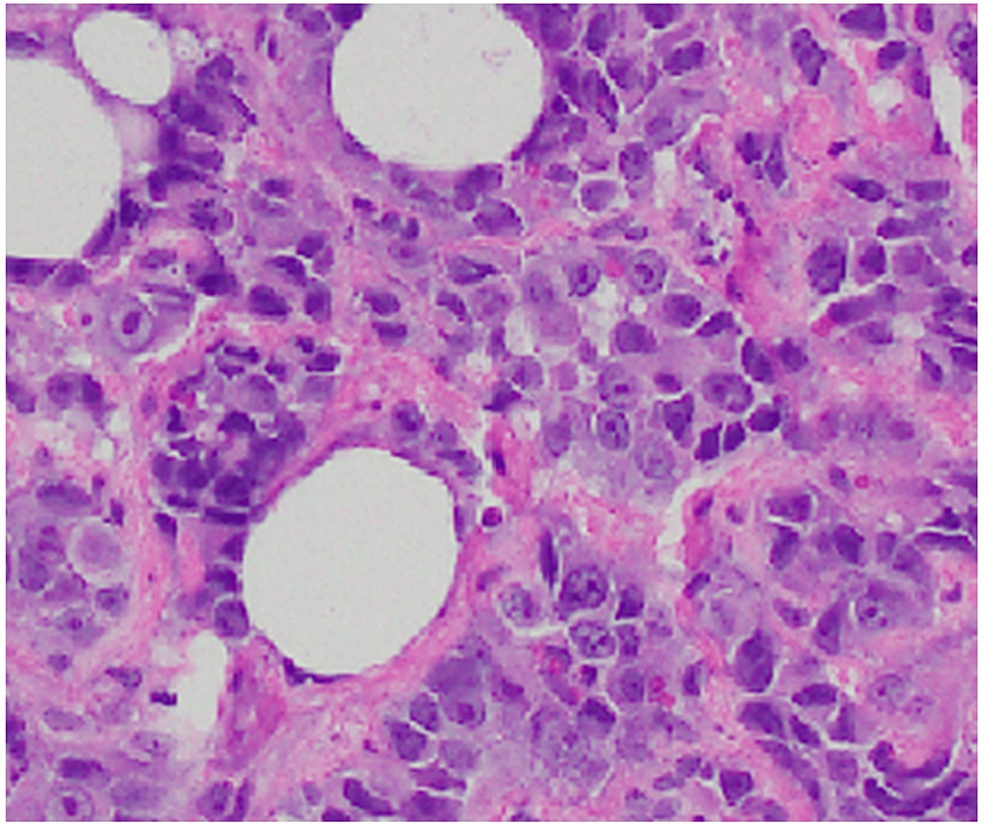
Histopathology of the punch biopsy Note the pleomorphic and hypercellular endothelial cells with entrapment of the collagen bundles. Hematoxylin and eosin stain at 200x magnification

Gram stains were positive for Providencia stuartii, Morganella morganii, and Proteus mirabilis. Herpes simplex virus polymerase chain reaction (PCR) was negative. Blood culture was positive for coagulase-negative Staphylococcus, although this was deemed to likely be a contaminant. However, based on the sensitivity profiles, ertapenem was started, and aztreonam, vancomycin, and acyclovir were discontinued. The patient had two punch biopsies and the histopathology specimens were congruent with angiosarcoma (Figure 3).

Immunohistochemistry (IHC) was performed, which demonstrated that the tumor cells were pleomorphic and diffusely stained with CD31/ ERG/P53, as well as partially positive for CD34. TFE3 was also positive so a differential included epithelioid hemangioendothelioma. However, the histopathological findings were more specific for angiosarcoma. Ki67 was performed for prognostic purposes and demonstrated 30% positivity.

Computed tomography (CT) abdomen and pelvis showed mild skin thickening noted in the lower right anterior abdominal wall. No significant fluid collection or subcutaneous emphysema was present. Additionally, reactive right inguinal lymphadenopathy was present (largest 2.3 cm). CT head showed chronic encephalomalacia in the right frontal + parietal lobe, with no evidence of metastases. Chest X-ray was notable for cardiomegaly with moderate left-sided pleural effusion and associated atelectatic changes, in keeping with her heart failure.

The patient will be followed with hematology/oncology service as an outpatient to receive treatment with paclitaxel and possibly resection if the tumor bulk decreases. For wound dressings, she will be receiving mupirocin ointment and wet-to-dry dressings.

## Discussion

Multiple risk factors for cutaneous angiosarcomas have been identified, including radiation, chronic lymphedema, and genetic syndromes. Environmental carcinogens have been primarily noted in hepatic angiosarcomas, but not cutaneous angiosarcomas, as the role of carcinogens and foreign bodies, such as tattoo ink, in this disease pathology is still unclear [3,4]. The patient outlined in this case report had no identifiable risk factors.

In prognostically unfavorable epithelioid angiosarcomas, the histological picture is dominated by “lawns” of large epithelioid cells, with narrow, blood-filled slits and fissures between [3]. However, notably, the degree of differentiation is not always inversely correlated with prognosis, like most other sarcomas [5]. On IHC, our patient had positive C31 and C34, which occur in 80% and 63% of cases, respectively. Other markers, which are frequently positive in cutaneous angiosarcoma, but not tested by the processing facility in this case, include factor VIII (83% positivity) and D2-40 (43% positivity) [3].

Angiosarcoma therapies depend on the stage of the disease itself. The National Comprehensive Cancer Network (NCCN) recommends surgical intervention for stage 1 angiosarcomas to ensure adequate oncologic margins [6]. For Stage II, IIIA, and IIIB diseases that can be resected with acceptable functional outcomes, the NCCN categorizes preoperative radiation therapy as a Category 1 recommendation. In the case of Stage IIIA-B disease, Category 2B recommendations include preoperative chemoradiation or chemotherapy, as well as postoperative chemoradiation. Therefore, a multimodal treatment approach with primary surgery followed by adjuvant radiotherapy and/or should be the goal in resectable tumors.

Due to the large area of body surface area (BSA) involvement (approximately 10%), advanced (presumed T4N1M0 staging), and multiple underlying comorbidities, the decision was made to obtain palliative chemotherapy with paclitaxel. Most publications on chemotherapy for inoperable angiosarcoma involve pegylated liposomal doxorubicin and paclitaxel. In the ANGIOTAX study with 30 patients, 80 mg/m^2^ paclitaxel was administered on days 1, 8, and 15 every four weeks, achieving a progression-free survival (PFS) of 74% after two months and 45% after four months [7]. Additionally, a large retrospective analysis of 125 cases showed very similar results for paclitaxel, with a median progression-free survival of 4.2 months [8].

## Conclusions

This manuscript describes a rare presentation (both in location and due to presentation with acute infection) of cutaneous angiosarcoma. The unusual presentation and rapid development of cutaneous angiosarcoma in this case will hopefully expand our understanding of different presentations of the disease and encourage providers to act expeditiously to improve the prognostic outcome. It will be of interest to readers in hospital medicine, family practice, hematology/oncology, infectious disease, and dermatology, as the case features multidisciplinary management involving each of these specialties. Additionally, the clinical images of the featured patient will add to the reservoir of dermatology images of patients with skin of color, in whom cutaneous angiosarcoma is underrepresented.
